# Ecological niche models display nonlinear relationships with abundance and demographic performance across the latitudinal distribution of *Astragalus utahensis* (Fabaceae)

**DOI:** 10.1002/ece3.6532

**Published:** 2020-07-08

**Authors:** Kathryn C. Baer, John L. Maron

**Affiliations:** ^1^ Anchorage Forestry Sciences Laboratory USDA Forest Service Pacific Northwest Research Station Anchorage AK USA; ^2^ Department of Biological Sciences University of Montana Missoula MT USA

**Keywords:** climatic suitability, ecological niche model, geographic distribution, stochastic population growth, suitability–abundance relationship, suitability–demography relationship

## Abstract

The potential for ecological niche models (ENMs) to accurately predict species' abundance and demographic performance throughout their geographic distributions remains a topic of substantial debate in ecology and biogeography. Few studies simultaneously examine the relationship between ENM predictions of environmental suitability and both a species' abundance and its demographic performance, particularly across its entire geographic distribution. Yet, studies of this type are essential for understanding the extent to which ENMs are a viable tool for identifying areas that may promote high abundance or performance of a species or how species might respond to future climate conditions. In this study, we used an ensemble ecological niche model to predict climatic suitability for the perennial forb *Astragalus utahensis* across its geographic distribution. We then examined relationships between projected climatic suitability and field‐based measures of abundance, demographic performance, and forecasted stochastic population growth (λ_s_). Predicted climatic suitability showed a J‐shaped relationship with *A. utahensis* abundance, where low‐abundance populations were associated with low‐to‐intermediate suitability scores and abundance increased sharply in areas of high predicted climatic suitability. A similar relationship existed between climatic suitability and λ_s_ from the center to the northern edge of the latitudinal distribution. Patterns such as these, where density or demographic performance only increases appreciably beyond some threshold of climatic suitability, support the contention that ENM‐predicted climatic suitability does not necessarily represent a reliable predictor of abundance or performance across large geographic regions.

## Graphical abstract

Estimates of climatic environmental suitability for the presence of a species derived from ecological niche models (ENMs) have been proposed as predictors of the abundance and demographic performance of that species across the landscape, but simultaneous tests of suitability–abundance and suitability–demography relationships across a species' geographic distribution are rare. In this study, we examined relationships between ENM‐derived climatic suitability and both the abundance and multiple metrics of demographic performance of a perennial forb across its latitudinal distribution. We found J‐shaped relationships between suitability and both abundance and overall demographic performance as measured by stochastic population growth rate, suggesting that ENM predictions of climatic suitability are not reliable indicators.

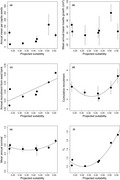

## 1 INTRODUCTION

A core aim of ecology and biogeography is to understand how environmental conditions influence the abundance or demographic performance of species across space. Improved insight regarding conditions that support higher abundance or demographic performance of species can in turn increase understanding of the factors constraining range limits and inform conservation efforts aimed at identifying sites capable of sustaining species of conservation concern (Araújo & Williams, 2000). Long‐standing biogeographical theory predicts that in the absence of density dependence, abundance and demographic performance of a species should peak at the center of its geographic distribution and decrease monotonically with proximity to the range boundary. This is theorized to be due to linear degradation of environmental suitability from the center to the edge of the range (the Abundant Center Hypothesis—*hereafter* ACH; Hengeveld & Haeck, 1982; Brown, 1984; Sexton, McIntyre, Angert, & Rice, 2009). However, empirical support for the ACH is weak (Abeli, Gentili, Mondoni, Orsenigo, & Rossi, 2014; Sagarin & Gaines, 2002; Sagarin, Gaines, & Gaylord, 2006; Sexton et al., 2009), suggesting that variation in environmental suitability for the occurrence and performance of a species is often more nuanced than can be explained by geographic position alone (Holt, 1997; Weber, Stevens, Diniz‐Filho, & Grelle, 2017). Rather than the generally inaccurate practice of using range position as a proxy for abundance and demographic performance, metrics of habitat suitability incorporating more realistic representations of geographical variation in environmental conditions could be used to more accurately predict density and demographic performance across the landscape (e.g. Tôrres et al., 2012; Van Couwenberghe, Collet, Pierrat, Verheyen, & Gégout, 2013; Thuiller et al., 2014; Matthiopoulos, Field, & MacLeod, 2019). This concept lies at the heart of the niche centroid hypothesis (Martinez‐Meyer, Diaz‐Porras, Peterson, & Yanez‐Arenas, 2013), which predicts that centrality in the sense of proximity to ideal niche conditions rather than geographic centrality is predictive of the abundance of a species across space. The niche centroid hypothesis posits that cases in which the abundance of a species is correlated with its proximity to the distribution's geographic center are a coincidental outcome of geographic centrality aligning with niche centrality.

One way in which niche centrality is estimated is through the use of ecological niche models (ENMs), which describe the environmental niche of a species in order to predict where suitable environmental conditions for its presence occur across the landscape. ENMs make predictions based on correlations between spatially explicit records of a species' presence (or presence and absence) and the environmental conditions at the location of the record (Elith et al., 2006; Peterson & Soberón, 2012). In other words, these models determine the environmental conditions that correspond to the presence of a species and use this information to predict suitability for its occurrence across the landscape. While newer approaches to distribution modeling may directly or indirectly incorporate predictors describing the presence of interacting species, dispersal, or indicators of nonclimatic environmental conditions such as disturbance (Bucklin et al., 2015; Engler et al., 2009; Linder et al., 2012; Randin, Vuissoz, Liston, Vittoz, & Guisan, 2009; Wisz et al., 2013), the majority of ENMs solely use large‐scale abiotic variables such as climate and topography as predictors of habitat suitability (Austin, 2007; Bucklin et al., 2015; Pearson & Dawson, 2003).

Species' distributions have often been described as a spatial manifestation of their niche (sensu Hutchinson, 1957), with the assumption that the processes which define and govern presence within niche space also determine the abundance and performance of the focal species within it (Holt, 2003). Patterns of occurrence, abundance, and demographic performance are often theorized to be linked, such that higher occupancy of a species in an area indicates higher habitat quality and thus higher abundance and demographic performance of the species (Holt, 1997; Gaston et al., 2000; Holt, Gaston, & He, 2002; *but see* Dallas & Hastings, 2018). The practice of utilizing ENM‐derived predictions of environmental suitability as an indicator of a species' abundance or performance assumes a relationship between projected suitability for presence and its local abundance (suitability–abundance relationship) or demographic performance (suitability–demography relationship) driven by the same environmental conditions associated with its presence. This assumption hinges upon the expectation that the environmental conditions which determine a species' presence similarly influence its abundance and demographic performance.

Although it has been claimed that the relationship between landscape‐scale occupancy and local abundance is among the most strongly supported in ecology (Holt et al., 2002), the ubiquity of suitability–abundance relationships remains a topic of substantial debate. On one hand, Weber et al. (2017) found support for a general pattern of positive abundance‐suitability relationships in meta‐analysis that examined 450 species spanning 30 studies. On the other hand, a recent analysis of the relationship between ENM‐predicted suitability and abundance of 396 mammal and tree species found no such relationship (Dallas & Hastings, 2018). The smaller number of studies that have evaluated the relationship between demographic performance and projected suitability yield similarly equivocal results: positive suitability–demography relationships have been observed in some cases (Brambilla & Ficetola, 2012; McLane & Aitken, 2012; Monnet, Hardouin, Robert, Hingrat, & Jiguet, 2015; Searcy & Shaffer, 2016; Sheppard, Burns, & Stanley, 2014), but appear to be the exception rather than the rule (Bacon et al., 2017; Bayly & Angert, 2019; Chardon, Pironon, Peterson, & Doak, 2020; Csergő et al., 2017; Oliver et al., 2012). The lack of consensus regarding the ubiquity of suitability–abundance and suitability–demography relationships may be due to local‐scale constraints not captured in the large‐scale predictors often used in ENMs (Davis, Jenkinson, Lawton, Shorrocks, & Wood, 1998; Guisan & Thuiller, 2005; Lembrechts, Nijs, & Lenoir, 2019; Pearson & Dawson, 2003; Varner & Dearing, 2014; Wisz et al., 2013), such that suitability predicts a maximum abundance or performance at a site that is then altered by attributes of the local environment (VanDerWal, Shoo, Johnson, & Williams, 2009).

Studies that examine the relationship between projected environmental suitability and a species' abundance *and* its demographic performance across its geographic distribution are rare. Those which do so tend to find contrasting patterns in suitability–abundance and suitability–demography relationships, and in that suitability is often positively correlated with abundance but not with population performance or estimated persistence (Oliver et al., 2012; Bean et al., 2014; Thuiller et al., 2014; *but see* Pironon, Villellas, Morris, Doak, & García, 2015). More studies of this kind are needed to further our understanding of whether ENM predictions of suitability might prove broadly useful for predicting species' abundance and performance across their distributions.

Here, we explore suitability–abundance and suitability–demography relationships across the latitudinal distribution of the perennial forb, *Astragalus utahensis*. Specifically, we ask whether predictions of climatic suitability derived from correlative ENMs accurately predict variation in the abundance and demographic performance of this species across its latitudinal distribution and what environmental predictors contribute most strongly to estimates of climatic suitability across the distribution.

## 2 MATERIALS AND METHODS

### 2.1 Study system

**Figure 1:**
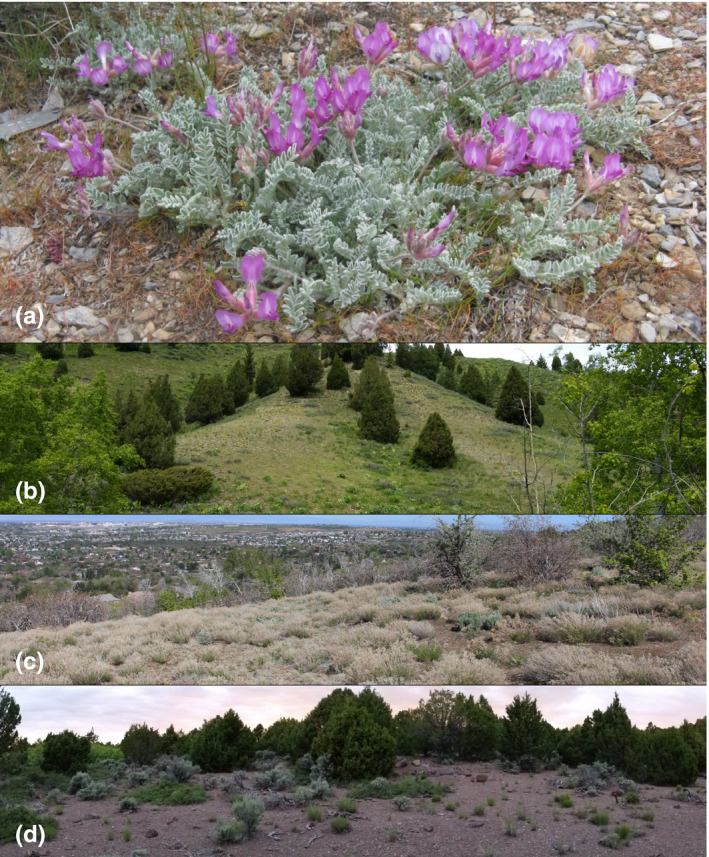
Study species and selected study sites. (a) *Astragalus utahensis* in flower, (b) the northern edge Reservoir site, (c) the central Uinta site, and (d) the southern Bull Spring site

**Figure 2:**
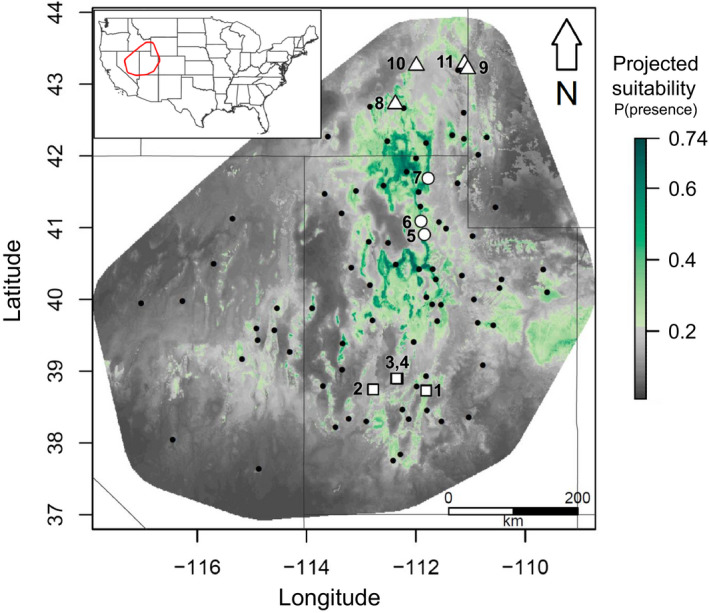
Map showing locations of thinned *A. utahensis* presence records included in the construction of ENMs (from herbarium collection records and field surveys conducted for this study, gray points), study populations (squares‐ southern edge populations, circles‐ central populations, and triangles‐ northern edge populations), and predicted suitability from the ensemble ENM. Gray indicates areas of predicted absence and green indicates areas of predicted presence, with darker gray representing lower suitability and darker green higher suitability

*Astragalus utahensis* occurs in spatially isolated populations on sparsely vegetated hillsides throughout its latitudinal distribution, which extends from southern Utah, USA (roughly 38°N) to southeastern Idaho, USA (roughly 43°N; Figures 1 and 2). It grows as a spreading basal rosette of villous compound leaves; the primary growing season is April through early July. Reproduction is solely by seed: plants produce racemes of 3–10 large magenta flowers which develop into hirsute seed pods containing medium‐large gravity‐dispersed seeds that fall in late June‐early July (Green, 1976). Although the distribution of *A. utahensis* is relatively small, abiotic conditions vary substantially across the latitudinal range. Climatic data (from 1970 to 2000) show higher mean annual temperatures in the center of the latitudinal range compared with its northern and southern edges and a gradient of high to low annual precipitation from the northern edge of the range to its southern edge (Worldclim v. 2.0; Fick & Hijmans, 2017; see Appendix S1).

To characterize the current distribution of *A. utahensis*, we obtained *A. utahensis* collection records from the databases of the Consortium of Pacific Northwest Herbaria, Herbarium of the New York Botanical Garden, the Stanley L. Welsh Herbarium Collection at Brigham Young University, the Utah State University Herbarium, the Utah Valley State College Herbarium, and the USDA Plants database. We removed the northernmost records in the dataset after confirming that the species appeared to be misidentified in these instances. We supplemented records with field observations of *A. utahensis* presence that were not already included in the set of herbarium records (Figure 2). We defined the center of the distribution as the latitudinal midpoint between the northernmost and southernmost recorded populations and identified three “central” study populations near this location (Figure 2; Table 1; populations 5–7). The four northern edge populations included in this study comprised three of the four northernmost populations included in herbarium records along with an additional extreme north population found during an extensive search along road corridors near these previously recorded populations (Figure 2; Table 1; populations 8–11). The “southern edge” study populations were not the furthest south within our query of herbarium records, as access to the remote areas represented by records at the extreme southern edge was deemed too difficult for logistical feasibility. Nonetheless, the four southern edge study populations included in our study were within 120 km of the furthest south recorded populations for this species (Figure 2; Table 1; populations 1–4).

**Table 1:** Locations and projected suitability of populations included in this study, including years in which each demographic rate was measured in each population

Range Position	Population Number	Site	Latitude	Longitude	Projected Suitability	Density	Per Capita Seeds	Basal Rosette Area & Growth	Survival	Germination	Stochastic *λ*
Southern Edge	1	Bull Spring	38.7302	−111.8136	0.343	2015, 16	2015	NA	NA	NA	NA
	2	South Twin Peak	38.7443	−112.7816	0.146	2015, 16	2015	NA	NA	NA	NA
	3	Meadow Creek 2	38.8965	−112.3387	0.153	2015, 16	2015	NA	NA	NA	NA
	4	Meadow Creek 1	38.8967	−112.3589	0.168	2015, 16	2015	NA	NA	NA	NA
Center	5	Bountiful	40.9025	−111.8472	0.560	2014, 16	2013–2016	2013–2016	2014–2016	2014–2016	2014–2016
	6	Uinta	41.0910	−111.9066	0.510	2014, 16	2013–2016	2013–2016	2014–2016	2014–2016	201420–16
	7	Providence	41.6903	−111.7742	0.229	2014, 16	2013–2016	2013–2016	2014–2016	2014–2016	2014–2016
Northern Edge	8	Nordic Center	42.7229	−112.3752	0.322	2014, 16	2013–2016	2013–2016	2014–2016	2014–2016	2014–2016
	9	Swan	43.2094	−111.0522	0.225	2014, 16	2013–2016	2013–2016	2014–2016	2015–2016	2014–2016
	10	Wolverine	43.2589	−111.9913	0.424	2014, 16	2013–2016	2013–2016	2014–2016	2014–2016	2014–2016
	11	Reservoir	43.2851	−111.1103	0.417	2014, 16	2013–2016	2013–2016	2014–2016	2014–2016	2014–2016

Note

Population number refers to numbered populations presented in Figure 2.

### 2.2 Ecological niche model construction

We removed duplicate records from herbarium records and field observations of *A. utahensis*, as well as records that were misidentified and those without at least two decimal degrees precision for latitude and longitude coordinates. This resulted in a final dataset of 106 presence records. Our sampling frame for the construction of ENMs was a polygonal convex hull that buffered presence points by 100 km. We used a sampling frame larger than the distribution of presence records because a previous study found that habitat suitable for the presence of *A. utahensis* exists more than 200 km beyond the northern edge of its current distribution (Baer & Maron, 2019).

An average nearest neighbor analysis revealed significant spatial autocorrelation between presence records within the sampling frame; we corrected for this by randomly thinning presence records to a minimum distance of 13 km between presence records (spatstat package; Baddeley & Turner, 2005). This thinning distance maximized the number of records retained while ensuring that average nearest neighbor distance between presence records was not significantly different from that of an equally sized sample randomly drawn from within the sampling frame (Monte Carlo simulation one‐sided *p = *.16). Of the 106 original presence records, 83 were retained post‐thinning. We built models using a dataset consisting of the 83 thinned presence records and 830 pseudoabsences (10 times the number of presences; 10*n*) selected randomly from fishnet cells within the study area that had not contained a presence record removed during the thinning process. Although Lobo and Tognelli (2011) espouse the use of 100*n* pseudoabsences, using 10*n* pseudoabsences yielded models with higher mean accuracy and is in keeping with methods advocated by Chefaoui and Lobo (2008).

Data describing climatic conditions from 1970 to 2000 associated with each presence and pseudoabsence record were queried from the Worldclim 2.0 dataset at a 30 arc‐second (~1 km) resolution and used as explanatory variables in ENMs (Fick & Hijmans, 2017). We selected a subset of climatic predictors for inclusion in the ENMs to eliminate multicollinearity among variables. To do this, we first performed univariate generalized linear models for *A. utahensis* presence using each climatic predictor, retained the variable with the greatest explanatory power, and discarded all variables correlated with it at |*r*| > 0.7. We repeated this process until no additional variables remained, leaving us with the following list of climatic predictors, all correlated at |*r*| ≤ 0.7: precipitation of the driest month (bio14), temperature seasonality and precipitation seasonality (bio4 and bio15, respectively), isothermality (bio3), and mean temperature of the coldest, wettest, and driest quarters (bio 11, bio 8, bio 9, respectively).

ENMs for *A. utahensis* were built using the biomod2 package (version 3.3–7.1; Thuiller, 2019) in R (version 3.5.3; R Core Development Team, 2019). To avoid bias associated with the use of a single model algorithm and leverage the strengths of multiple algorithms (Araújo & New, 2007), we utilized an ensemble modeling approach. Ensemble models comprised a total of six algorithms: three regression algorithms and three machine learning methods. Regression algorithms included (a) generalized linear models (GLM; McCullagh & Nelder, 1989), (b) generalized additive models (GAM; Hastie & Tibshirani, 1986, 1990), and (c) multivariate adaptive regression splines (MARS; Friedman, 1991). Machine learning algorithms included (a) maximum entropy modeling (MAXENT; version 3.4.1; Phillips, Anderson, & Schapire, 2006), (b) random forest (RF; Breiman, 2001), and (c) boosted regression trees (BRT; De'ath, 2007; Elith, Leathwick, & Hastie, 2008). We chose these algorithms because of their high predictive accuracy among ENM algorithms (Cutler et al., 2007; Elith et al., 2006).

We performed 10 runs of each algorithm; each model run was trained on a random selection of 90% of presences and pseudoabsences and internally cross‐validated with the remaining 10% of presence and pseudoabsence data. We evaluated the accuracy of each model run using the area under the curve of the receiver operating characteristic (AUC; Hanley & McNeil, 1982) and the true skill statistic (TSS; Allouche, Tsoar, & Kadmon, 2006). The value of AUC ranges from 0 to 1, with AUC = 1 representing a perfect prediction and AUC ≥ 0.7 a useful prediction (Swets, 1988). TSS is similar to Cohen's Kappa (Cohen, 1960) but is less sensitive to prevalence (Allouche et al., 2006). TSS ranges from −1 to 1, where TSS < 0 indicates a prediction no better than random, TSS = 1 indicates perfect prediction and thresholds for evaluating model performance mirror those for Cohen's kappa, with a TSS value of ≥0.4 representing a useful model (Allouche et al., 2006). Runs of each algorithm for which TSS was greater than 0.45 were retained in the construction of the ensemble model. As a result, the unweighted mean AUC, TSS, sensitivity, and specificity values for internal cross‐validation of individual model runs were on average lower than those of the ensemble model. The AUC, TSS, sensitivity, and specificity values of each model run, along with a summary of the models included in the construction of the ensemble model, are presented in Table S2.1. The relative contribution of each model run to ensemble model predictions was a function of its TSS value relative to that of other retained model runs; these weights are presented in Table S2.1. We used the ensemble model to create prediction maps for suitability throughout the study area and extracted suitability predictions for each study site.

We determined the relative contribution of each variable to ensemble model predictions by calculating normalized importance values for all predictors in the ensemble model. Importance values for each variable were determined by comparing the correlation between the ensemble model prediction using the original data and the prediction using randomly drawn data and subtracting this value from one (Thuiller, 2019). Randomization of variables with high importance values has a large impact on model predictions, while randomization of less important variables affects the predictions proportionally less.

### 2.3 Density and demographic monitoring

We characterized the abundance of *A. utahensis* by measuring plant density in each study population. To do this, we placed multiple transects at randomly selected locations through each study population and quantified the density of *A. utahensis* in 1 m^2^ quadrats placed every 2 m on alternating sides of each transect. We measured density at the time of peak flowering in each population (late May‐early June). We recorded the number of individuals in each quadrat belonging to each of three life stages: seedlings (plants with identifiable cotyledons that had germinated in the year of the survey), juveniles (plants that had neither cotyledons nor developing or flowering racemes), and reproductive adults (plants with developing, flowering, or fruiting racemes). Censuses of plant density were conducted in central and northern edge populations in 2014 and 2016, and in southern edge populations in 2015 and 2016 (Table 1). Differences in reproductive and vegetative plant density among populations surveyed in different years likely reflect true density differences rather than year effects because annual juvenile and adult survival is high, recruitment is not highly episodic, and models of reproductive and juvenile plant density among sites do not show a significant variation among years (K.C. Baer and J.L. Maron; unpublished data). Therefore, we compared mean density across the first and second censuses for reproductive plants, juvenile plants, and their combined density among range locations even though the first census was performed in different years for some populations (2014 in northern and central populations, 2015 in southern populations). As seedling germination and resultant density are likely to vary more substantially depending upon temporal changes in germination cues, comparisons incorporating seedling density were only made when populations were surveyed during the same growing season.

In addition to performing surveys of plant density, we also evaluated aspects of plant demographic performance in each study population in 2015 (collection dates for abundance and demographic data are summarized in Table 1). In southern edge populations, we measured the basal rosette area (*hereafter*, size) of 30 juvenile and 40 reproductive plants (except the Meadow Creek 1 population, in which only 17 juvenile plants could be located) according to the formula for the area of an ellipse. While not itself a demographic parameter, size is positively correlated with survival, growth, and reproduction in *A. utahensis* (Baer & Maron, 2019). We also estimated the per capita seed production of the reproductive plants. To do this, we recorded the total number of fruits produced by the 40 reproductive adult plants that had been measured. If a reproductive plant produced fruits (some plants aborted all developing fruits), up to 10 fruits were collected. If fewer than 20 of the 40 measured reproductive plants had produced fruits, we sampled fruits from additional reproductive plants until we had collected fruits from 20 plants. An exception was the Meadow Creek 1 population, in which only 7 reproductive plants in the population had produced fruits. We counted the seeds in each collected fruit to assess the mean seeds per fruit for each plant and multiplied this value by the total number of fruits on that individual to estimate per capita seed production. In study populations at the center and northern edge of the distribution, we collected sufficient demographic data from 2013 to 2016 to build size‐based integral projection models for stochastic population growth rate (*λ*_s_; *methods described in* Baer & Maron, 2019). Included in these demographic data were annual measures of the size and growth of 40–60 juvenile plants and 30–35 reproductive plants, per capita seed production of the 30–35 reproductive plants, annual survival of the measured juvenile and reproductive plants and 60–80 marked seedlings, the size of seedlings that survived from one growing season to the next, and both cumulative germination rates over the course of 3 years and rates of germination from the seed bank in the final year of a 3‐year seed addition study. As the northern edge Swan population was added to the study in 2014, it was excluded from analyses of cumulative germination (which required 3 years of data) but included in analyses of germination from the seed bank.

### 2.4 Statistical analyses

All statistical analyses were performed using R Statistical Software (version 3.5.3; R Core Development Team, 2019). We used linear regressions to test for linear and exponential relationships between ENM‐predicted climatic suitability (*hereafter,* suitability) and (a) log‐transformed seedling density and log‐transformed overall plant density (seedlings, juvenile plants, and reproductive plants) in 2016 only, as this was the only year in which all populations were visited, (b) log‐transformed mean density of reproductive plants, juvenile plants, and the sum of these values across the two censuses conducted in each study site, and (c) mean plant size and (d) log‐transformed per capita seed production, which were only measured in all southern, central, and northern populations in 2015. Exponential terms were removed from models where they were not at least marginally significant. Log‐transformation of density and per capita seed production was used to normalize the right‐skewed distributions of each set of data.

Using the expanded set of demographic data collected from 2013 to 2016 in central and northern edge populations (2014–2016 in the Swan population), we tested for significant linear and exponential relationships and between suitability and (a) log‐transformed per capita seed production, (b) basal rosette growth (square‐root transformed to account for the response's right‐skewed distribution that included negative values), (c) the size of surviving seedlings, and (d) annual survival across years. To do this, we used repeated‐measures generalized linear mixed models (GLMMs; lme4, Bates, Mächler, Bolker, & Walker, 2015; lmerTest, Kuznetsova, Brockhoff, & Christensen, 2017) with suitability, suitability^2^, measurement year, and interactive suitability*measurement year and suitability^2^*measurement year terms included as fixed effects and study site as a random effect. Exponential terms were removed from models where they were not at least marginally significant. We used linear regressions to test for linear and exponential relationships between suitability and (a) cumulative germination over three years, (b) germination rates from an experimentally established seed bank, and (c) model‐derived estimates of *λ*_s_ in northern and central study populations. These tests allowed us to examine additional demographic transitions that might underlie suitability–abundance or suitability–demography relationships in central and northern populations and speculate about the transitions that may drive these relationships in southern populations. We used a linear regression to examine the relationship between log‐transformed mean overall plant density and *λ*_s_ in northern and central study populations.

## 3 RESULTS

### 3.1 Ecological niche model

The ensemble model was highly accurate (AUC = 0.97, unweighted mean of component models = 0.78 ± *SD* 0.07; TSS = 0.81, unweighted mean of component models = 0.57 ± *SD* 0.08). Model sensitivity (accurate prediction of presences) was 0.93 (unweighted mean of component models: 0.88 ± *SD* 0.08), and specificity (accurate prediction of pseudoabsences) was 0.88 (unweighted mean of component models: s0.69 ± *SD* 0.11). The relative importance of all predictors included in the models, along with their relationships with ensemble model suitability predictions, are presented in Appendix S2. In brief, precipitation in the driest month (bio14) was the most important predictor of suitability in the ensemble model, followed in importance by temperature seasonality (bio4), with a general pattern of higher suitability in areas of higher annual variation in temperature (Figure S2.1).

SDM predictions for the suitability of study populations ranged from 0.153 to 0.560, with generally high suitability scores near the center of the latitudinal and longitudinal range, although the western foothills of the Wasatch Mountains were also predicted to be highly suitable across latitudes (Figure 2). Suitability scores showed a significant quadratic relationship with latitude (Table 1; K.C. Baer and J.L. Maron, unpublished data). Peripherally outlying populations, particularly along the western and southwestern range edges, were often in the areas of lower suitability, although the ensemble ENM predicted small areas of high suitability near the southern, eastern, and northern boundaries of the study area. Furthermore, a large continuous area of high suitability was projected beyond the eastern range edge, and smaller areas of high suitability were projected beyond the northern extent of the current range (Figure 2).

### 3.2 Suitability, density, and demography

**Figure 3:**
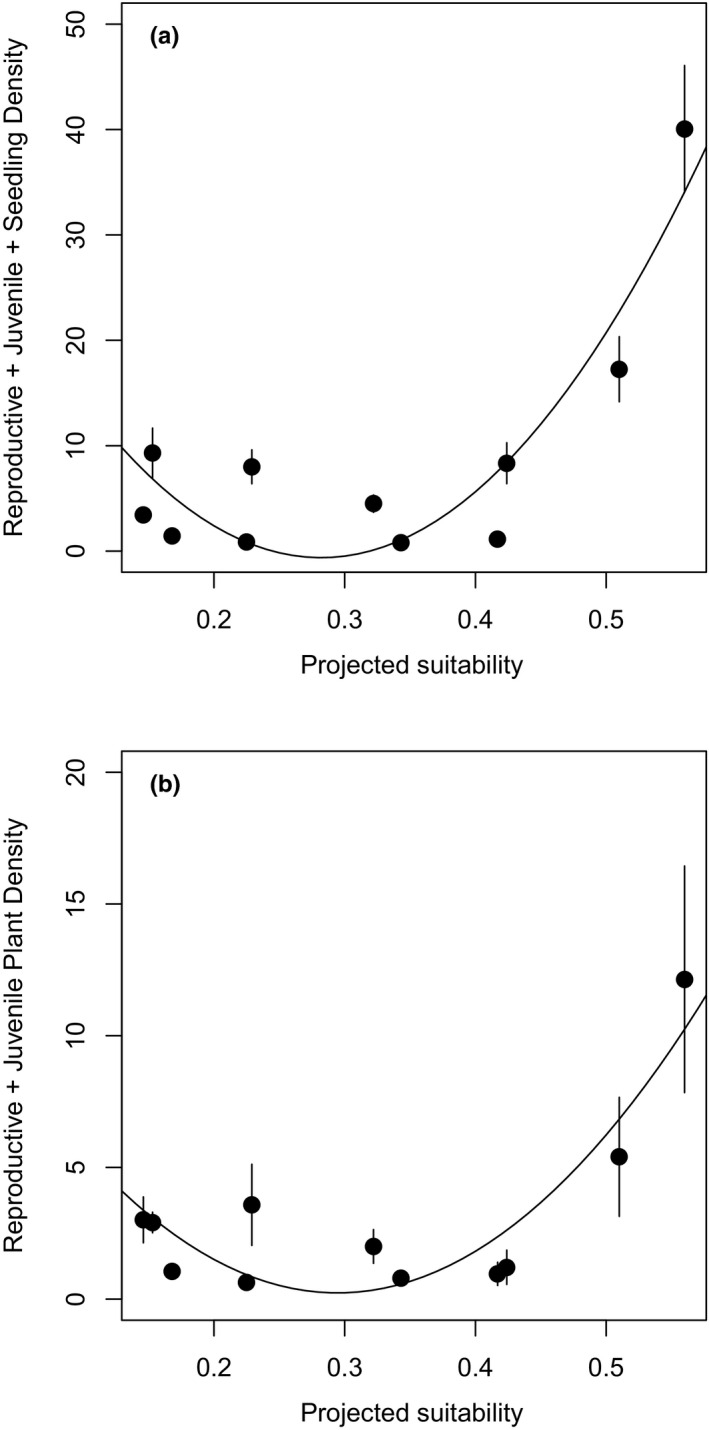
Relationship between ensemble ENM‐predicted suitability and mean ± *SEM* (a) overall plant density (reproductive + juvenile + seedling) during the 2016 census, and (b) summed reproductive and juvenile plant density across the first and second censuses in southern edge, central, and northern edge sites

The log‐transformed summed density of all life stages in 2016 and mean summed reproductive and juvenile plant density showed a significant J‐shaped relationship with suitability (Figure 3; Table 2). Log‐transformed seedling density in 2016 exhibited a marginally significant J‐shaped relationship with suitability (Table 2).

**Table 2:** Outcomes of repeated‐measures linear regressions and GLMMs of *A. utahensis* density and demographic rates versus ENM‐predicted suitability, study year, and the interaction of suitability and year (where applicable)

	Suitability			Suitability^2^		
	*df*	*F*/*χ* ^2^	*p*	*df*	*χ* ^2^	*p*
Southern, Central, and Northern Sites						
ln (Overall Density) (2016 only)	1, 8	2.57	.15	1, 8	6.16	**.04**
ln (Seedling Density) (2016 only)	1, 8	3.96	.08^†^	1, 8	4.91	.06^†^
ln (Reproductive + Juvenile Plant Density)	1, 8	3.08	.12	1, 8	10.88	**.01**
ln (Reproductive Plant Density)	1, 8	3.87	.08	1, 8	17.23	**.003**
ln (Juvenile Plant Density)	1, 8	1.69	.23	1, 8	3.80	.09^†^
Basal Rosette Area	1, 9	0.33	.58			
ln (Per Capita Seeds)	1, 9	1.36	.27			

	Suitability			Suitability^2^			Census Year			Suitability* Census Year			Suitability^2^* Census Year		
	*df*	*F*/*χ* ^2^	*p*	*df*	*F*/*χ* ^2^	*p*	*df*	*χ* ^2^	*p*	*df*	*χ* ^2^	*p*	*df*	*χ* ^2^	*p*
Central and Northern Sites Only															
ln (Per Capita Seeds)	1	1.71	.19				2	1.52	.47	2	1.07	.59			
Cumulative Germination (2014–2016)	1, 3	4.96	.11	1, 3	24.78	**.02**									
Germination from Seed Bank	1, 5	8.36	**.03**												
sqrt(Basal Rosette Growth)	1	0.28	.60				2	8.36	**.02**	2	4.44	.11			
ln (Basal Rosette Area: Surviving Seedlings)	1	2.13	.14				2	4.55	.10	2	1.39	.50			
Annual Survival (All Life Stages)	1	3.54	.06^†^	1	4.38	**.04**	2	4.60	.10	2	4.03	.13	2	4.30	.12
Stochastic Population Growth Rate (λ_s_)	1, 4	77.73	**<.001**	1, 4	37.90	**.004**									

Note

Bolded values indicate significance at *p* ≤ .05; marginal significance at *p* ≤ .1 is indicated by †.

**Figure 4:**
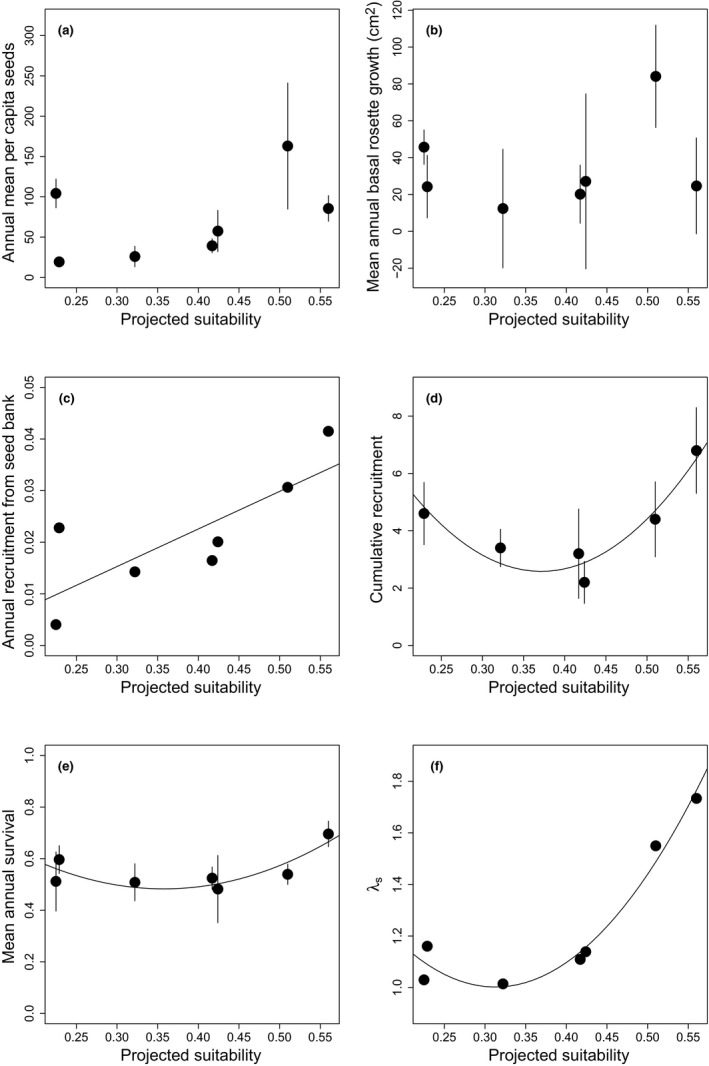
Relationship between ensemble ENM‐predicted suitability in central and northern study sites only and mean (±*SEM*) (a) annual per capita seed production, (b) cumulative recruitment from 250 seeds over 3 years of a seed addition study, and (c) annual survival, (d) annual basal rosette growth rate, (e) mean recruitment rate from the seed bank in the final year of a seed addition study, and (f) model‐estimated stochastic population growth rate (*λ*_s_). Regression lines indicate statistically significant relationships at *p* ≤ .05

Neither the basal rosette area nor the log‐transformed per capita seed production of plants in southern, central, and northern populations in 2015 was correlated with suitability in those populations. When measured solely in central and northern edge populations from 2013 to 2016, per capita seed production, basal rosette growth, and the size of surviving seedlings were also not correlated with suitability (Figure 4a,b; Table 2). Germination rates from the seed bank showed a positive linear relationship with suitability (Figure 4c; Table 2). J‐shaped relationships existed between suitability and cumulative recruitment over 3 years, annual survival, and λ_s_ of the central and northern edge study populations (Figure 4d–f; Table 2). Log‐transformed mean overall plant density across the 2014 and 2016 censuses had a positive linear relationship with λ_s_ in central and northern study populations (*F*_1, 5_ = 15.7, *p* = .01).

## 4 DISCUSSION

Across the entire latitudinal range, climatic suitability was not a strong predictor of *A. utahensis* density or demographic performance. At low‐to‐intermediate suitability, *A. utahensis* density was uniformly low, but then increased with increasing suitability scores beyond a particular threshold value. While demographic rates in central and northern edge populations showed a variety of relationships to suitability ranging from nonexistent to linear to exponential, the relationship between suitability and overall demographic performance across the center‐north portion of the distribution was quite similar to that between suitability and abundance across the entire latitudinal distribution. These results suggest that *A. utahensis* abundance and performance are also only influenced by climatic suitability beyond some threshold.

### 4.1 *Suitability* versus* abundance*

The lack of a linear suitability–abundance relationship across *A. utahensis*' latitudinal distribution contrasts with the findings of several previous studies which have demonstrated positive suitability–abundance relationships across taxonomic groups (Weber et al., 2017; e.g. invertebrates: Jiménez‐Valverde, Diniz, Azevedo, & Borges, 2009; Gutiérrez, Harcourt, Díez, Gutiérrez Illán, & Wilson, 2013; herbaceous vascular plants: Van Couwenberghe et al., 2013; trees: Thuiller et al., 2014; vertebrates: Russell et al., 2015). However, suitability–abundance relationships are generally weaker in plants than animals, and evidence suggesting that the strength of these relationships is unaffected by the proportion of the range examined is derived solely from studies of mammals and birds (Weber et al., 2017). Studies that have examined suitability–abundance relationships in plants often build ENMs using occurrence data collected according to a political boundary rather than throughout a species' entire distribution, which may yield biased conclusions regarding the ubiquity of observed relationships across the distribution (e.g. Van Couwenberghe et al., 2013; Thuiller et al., 2014).

Consistent with our results, a recent analysis of 246 mammal and 158 tree species found no clear relationship between SDM‐derived estimates of habitat suitability and species abundance (Dallas & Hastings, 2018). Where linear suitability–abundance relationships are weak or nonexistent, this may be attributable to: (a) differences in the identity or scale of climatic conditions that dictate presence compared to abundance (Boulangeat, Gravel, & Thuiller, 2012), (b) a stronger impact of disturbance and land‐use history (unrelated to overarching climatic conditions) on abundance (Jiménez‐Valverde et al., 2009; Nielsen, Johnson, Heard, & Boyce, 2005), (c) the influence of local biotic interactions on demographic performance and resultant density (Cabral & Kreft, 2012; Davis et al., 1998; Guisan & Thuiller, 2005; Linder et al., 2012), or (d) variation in dispersal ability that allows persistence in low‐suitability areas but prevents the colonization of highly suitable areas (Guisan & Thuiller, 2005; Pulliam, 2000; Van Horne, 1983).

Few of these potential explanations for the lack of a suitability–abundance relationship apply to *A. utahensis*. For example, while *A. utahensis* is dispersal limited beyond its northern range boundary (Baer & Maron, 2019), this does not explain the pattern of low density in populations of intermediate suitability that yielded a J‐shaped rather than linear suitability–abundance relationship. Moreover, *A. utahensis* generally grows in sparsely vegetated areas where negative intraspecific density dependence is rare or absent, although interspecific competition may impact density by excluding the species from more benign microsites in some populations (K.C. Baer, *personal observation*). Predispersal seed predation and pollen limitation decrease the demographic performance (and presumably the density) of *A. utahensis*, but the extent to which this occurs is similar across populations experiencing varying climatic suitability (Baer & Maron, 2018).

One potential explanation for the observed pattern is that the climatic conditions associated with the suitability of an area for the presence of *A. utahensis* may have relatively little impact upon its local abundance compared with other aspects of the climate in some areas of the distribution due to local adaptation, which could yield a suitability–abundance relationship such as the one we observed (*e.g*. Peterson, Doak, & Morris, 2019). For example, the climatic conditions that most strongly constrain *A. utahensis*' abundance could differ substantially in different parts of its latitudinal range. It is possible that the conditions contributing most strongly to ENM predictions of suitability for the presence of *A. utahensis* also play a large role in determining its abundance in central populations (two of the three highest‐abundance populations were central populations), but are of relatively minor importance relative to other climatic conditions in other portions of the range. A second possibility is that the observed suitability–density relationship could simply be an artifact of sampling bias: occurrence records could be more numerous where populations are larger and denser, resulting in higher suitability scores for these more visible high‐abundance populations. If bias in the number of presence records only appeared beyond a particular threshold of density, suitability may only exhibit a positive correlation with density once that threshold is reached. A third explanation for the consistently low density of populations below a particular suitability threshold may be that abundance simply does not respond strongly to climatic variation until some threshold of climatic favorability is met, regardless of the conditions that define a favorable climate in a particular region.

The expectation of a linear suitability–abundance relationship assumes that abundance of a species is positively related to its proximity to the “niche center” (as approximated by ENM suitability), much as the abundant center hypothesis predicts abundance to be tied to geographic centrality. Although we did not observe the linear relationship between a proxy for niche centrality (suitability) and abundance, the J‐shaped relationship between density and suitability was echoed by a strong correlation between density and the latitudinal position of study populations, with higher density in central than peripheral study sites characteristic of an abundant center distribution (K.C. Baer and J.L. Maron unpublished data). This suggests that estimates of suitability derived from our ensemble ENM (aka niche centrality) are no better a predictor of *A. utahensis* density across the distribution than the latitudinal position of a study population. This may be because our ensemble model does not adequately describe the entirety of *A. utahensis*' climatic niche. The ensemble model's specificity value indicates an absence was predicted where a presence had been recorded in roughly twelve percent of cases, meaning that it may often underestimate suitability for this species. Furthermore, evidence of dispersal limitation in *A. utahensis* (Baer & Maron, 2019) suggests that the current distribution may not fully reflect the range of environmental conditions suitable for its presence and that estimates of suitability based upon climatic conditions within the current distribution may be somewhat biased.

### 4.2 *Suitability* versus* demographic performance*

While some of the demographic parameters we examined were not related to predicted suitability, several important demographic rates did have significant relationships with suitability. The J‐shaped relationship between *λ*_s_ and the suitability of central and northern edge study populations appears attributable to a combination of similar relationships in cumulative recruitment and mean annual survival and a positive linear relationship between recruitment rates from the seed bank and suitability. We previously found that significant declines in *λ*_s_ between central and northern edge populations were primarily attributable to three demographic rates, of which germination rates from the seed bank and total seed production at the population scale were two (Baer & Maron, 2019). While per capita seed production was not significantly correlated with the suitability of central and northern edge study sites, reproductive plant density was positively associated with suitability in those sites, which likely led to increased total seed production at the population scale in more suitable populations (K.C. Baer and J.L. Maron, unpublished data). It seems likely that the relationship between *λ*_s_ and suitability is driven primarily by differences in reproductive plant density and germination from the seed bank with smaller contributions from annual survival and overall recruitment rates.

The J‐shaped nature of the relationship between suitability and *λ*_s_ in these populations in central and northern populations and strong positive correlation that we observed between overall *A. utahensis* density and *λ*_s_ in these populations, coupled with the J‐shaped suitability–density relationship across all southern‐northern study populations suggests that a similar J‐shaped relationship exists between suitability and *λ*_s_ across the entire latitudinal distribution. Such a relationship between suitability and demographic performance would indicate that ENM predictions of suitability cannot be directly translated into predictions for the performance of a species across its geographic distribution, as the threshold beyond which demographic performance responds strongly to suitability is not known a priori.

Previous studies that have examined *both* suitability–abundance and suitability–demography relationships typically find that the former are significant while the latter are not (Bean et al., 2014; Oliver et al., 2012; Pironon et al., 2015; Thuiller et al., 2014). Similarly, studies of solely suitability–demography relationships commonly find that suitability is not correlated with overall demographic performance in any consistent manner (Bacon et al., 2017; Bayly & Angert, 2019; Csergő et al., 2017; Pironon et al., 2018). As with explanations for a lack of a suitability–abundance relationship, the lack of a suitability–demography relationship is generally attributed to the influence of local conditions that yield habitat suitability which differs from climatic suitability. These may include heterogeneity in a site's local physiography, the availability of necessary resources unrelated to climate, or the distribution of facilitative partners, competitors, or predators, all of which may be obscured by single‐species SDMs built using solely large‐scale climatic predictors (e.g. Bean et al., 2014; Bayly & Angert, 2019). In addition, suitability based upon a model built for the entire distribution may not reflect the climatic optimum for all populations and may poorly predict demographic responses to climate variation across the distribution (Chardon et al., 2020; Peterson et al., 2019).

As with the suitability–density relationship, the nonlinear suitability–*λ*_s_ relationship we observed in central and northern populations and similar relationship predicted to exist across the entire latitudinal distribution may be attributed to differences in the environmental attributes that most strongly constrain demographic performance among geographic regions. For example, variation in microsite conditions in populations at the southern edge of the distribution may exert stronger control over density and demography in those populations than do larger‐scale climatic conditions. This possibility is supported by the fact that three of the four southern edge populations were located in an ephemeral stream bed (South Twin Peak) or beside an irrigation canal (Meadow Creek 1 & 2), which may have led these populations to perform better than predicted by large‐scale climatic conditions if the sites in which they occurred were less water‐limited than the surrounding landscape. According to the model's suitability threshold for presence of the species (0.203), the suitability of these areas was predicted to be too low for the presence of the species, so supplemental water availability may account for their existence and persistence.

The relatively poor demographic performance of populations with intermediate levels of predicted suitability (of which most were northern edge populations) may indicate that some aspect of the local environment unrelated to climate suppresses demographic performance to a level lower than might be anticipated if the suitability–demography relationship was linear. While *A. utahensis* tends to grow in sites that support few other species, it is possible that superior competitors in northern edge populations pushed the plants into even less suitable microsites than they inhabit at either low or high suitability.

The shape of the suitability–*λ*_s_ and suitability–density relationships that we observed was due in large part to high demographic performance and density in two high suitability populations near the center of the distribution (Uinta and Bountiful). As with density, it may be that demographic performance is uniformly poor below a certain threshold of climatic suitability, but responds strongly to increasing suitability beyond this threshold. If Uinta and Bountiful populations existed in areas where climatic conditions exceeded this threshold, this may have allowed them to flourish and help explain the resulting patterns we observed between suitability and both density and overall demographic performance in *A. utahensis*.

## 5 CONCLUSION

In this study, we found that projected climatic suitability displayed a J‐shaped relationship with the abundance of a species across its geographic range and a similar relationship with overall demographic performance as measured by stochastic population growth rate across the center to north portion of the range. A significant correlation between local density and population growth rate in central and northern edge study populations suggested that the relationship between suitability and population growth rate across the entire latitudinal distribution would be similar to that between suitability and density, meaning that demographic performance may only respond to increasing suitability beyond a threshold that is not known in advance. This implies that suitability is not a reliable metric for estimating either the abundance or demographic performance of a species across its geographic distribution.

## CONFLICT OF INTEREST

None declared.

## AUTHOR CONTRIBUTIONS

**Kathryn C. Baer:** Conceptualization (equal); data curation (lead); formal analysis (lead); funding acquisition (equal); investigation (lead); methodology (lead); project administration (lead); validation (lead); visualization (lead); writing–original draft (lead); writing–review and editing (lead). **John L. Maron:** Conceptualization (equal); formal analysis (supporting); funding acquisition (equal); methodology (supporting); visualization (supporting); writing‐original draft (supporting); writing‐review and editing (supporting).

### Open Research Badges

This article has earned an Open Data Badge for making publicly available the digitally‐shareable data necessary to reproduce the reported results. The data is available at https://osf.io/5ga32/.

## Supporting information

Supplementary Material

## Data Availability

Data and R scripts associated with this study are publicly available through the Open Science Framework digital repository at https://osf.io/5ga32/.
