# Supplementary material for: Ecological niche models display nonlinear relationships with abundance and demographic performance across the latitudinal distribution of Astragalus utahensis (Fabaceae)
Source: Ecol Evol. 2020 Jul 8;10(15):8251–64. doi: 10.1002/ece3.6532 (PMC7417238; doi:10.1002/ece3.6532)
Supplement: Supplementary file 1 — Supplementary Material [file ECE3-10-8251-s001.docx]

SUPPLEMENTAL MATERIALS

Appendix S1: Selected climatic variables for each study site and the study area.

Table S1.1: Values of bioclimatic variables included in component algorithms of the ensemble ENM for each study site.

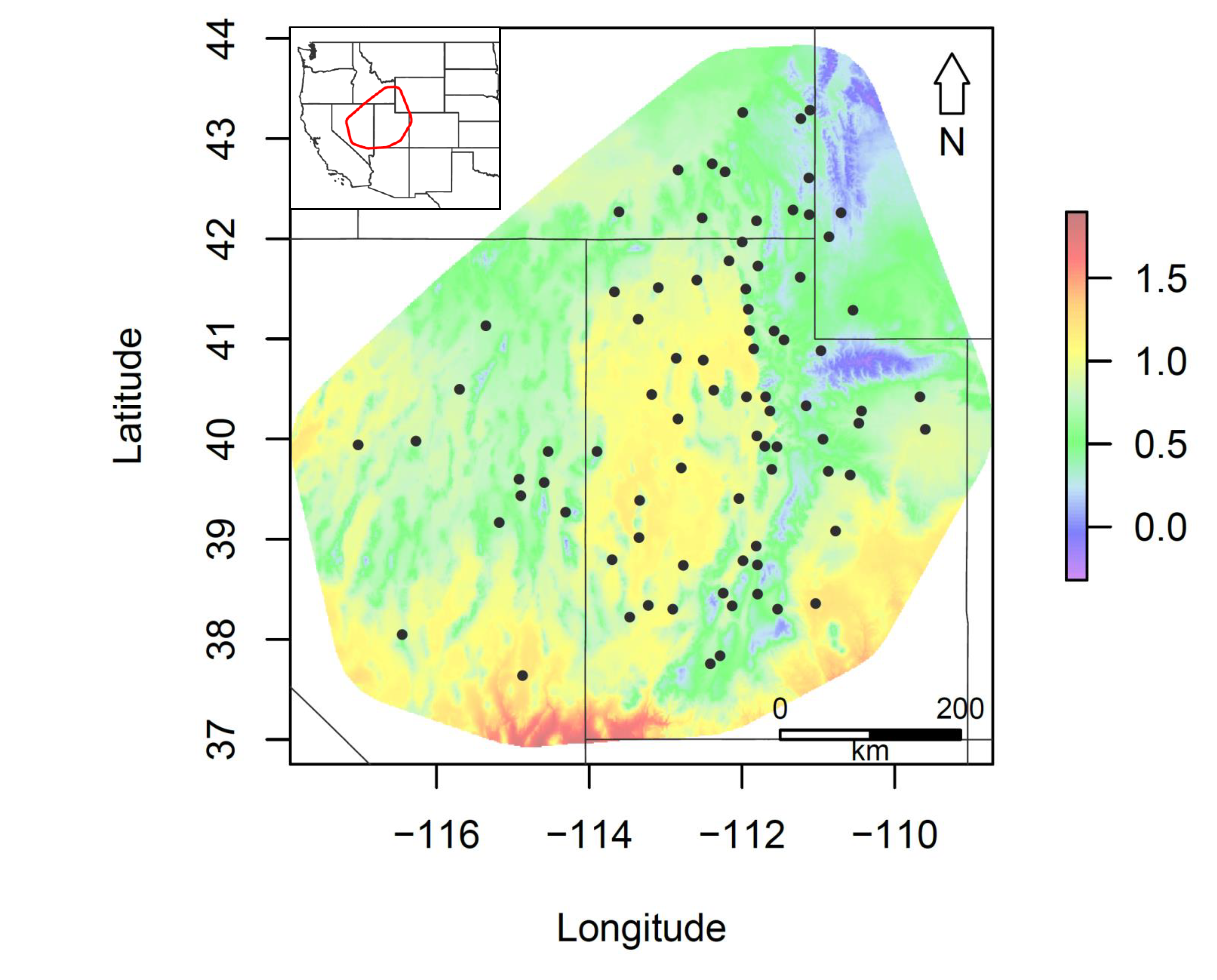


Figure S1.1: Mean annual temperature (°C) across the study area and locations of herbarium collection records and field study sites (black points).


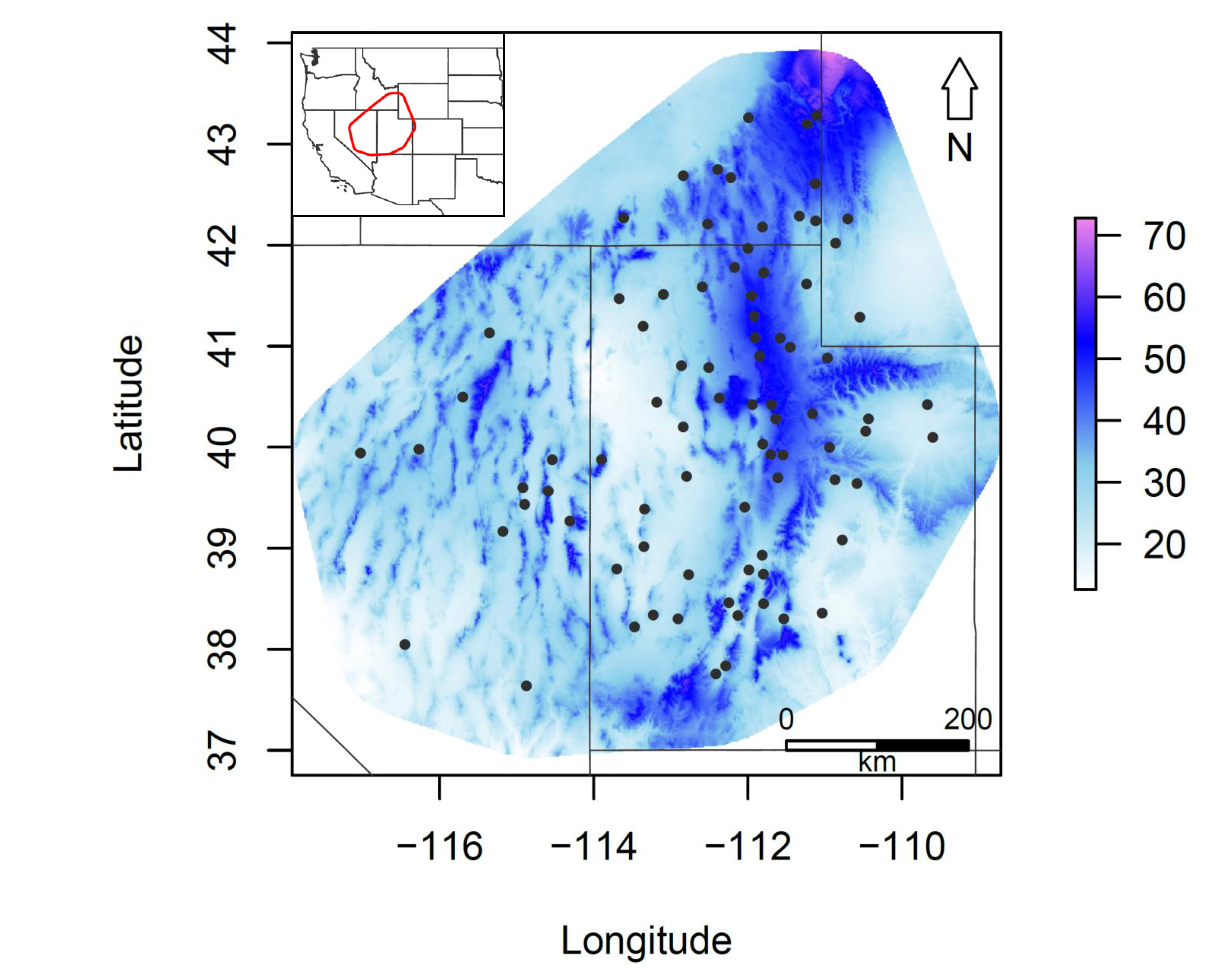


Figure S1.2: Mean annual precipitation (cm) across the study area and locations of herbarium collection records and field study sites (black points).

Appendix S2: Ensemble ENM outcomes.

Table S2.1: Normalized importance values for each bioclimatic predictor in all component models and the ensemble model.

Table S2.2: Accuracy metrics for all runs of each model algorithm. Model runs included in the ensemble model are marked with an asterisk and their relative weight in the ensemble model’s weighted average of component model predictions is indicated on a scale of 0 to 1.

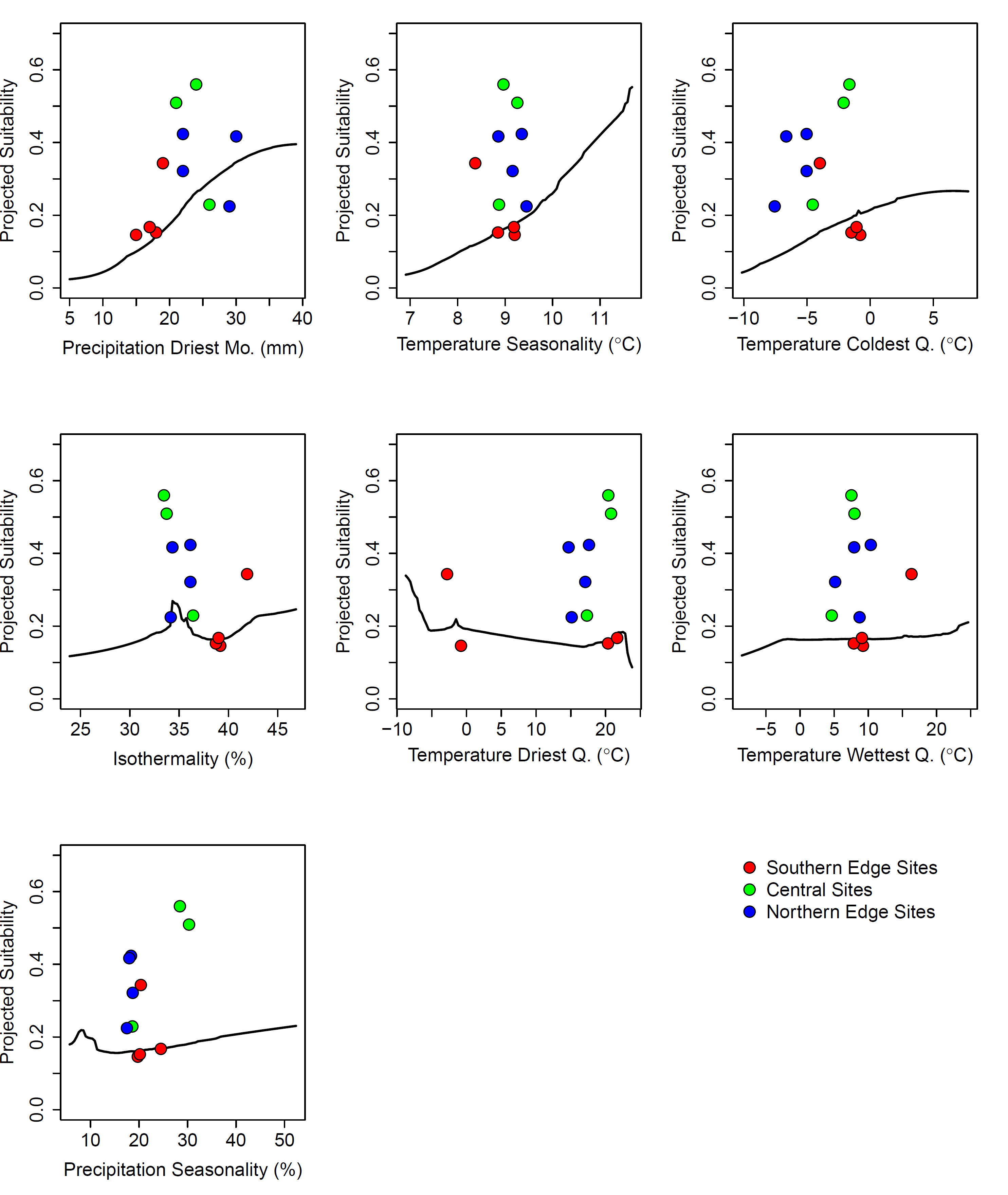


Figure S2.1: Response plots for the relationship between bioclimatic predictors and ensemble ENM suitability projections. Circles represent the value of each bioclimatic predictor and predicted suitability for each site. Red points indicate southern edge sites, green indicate central sites, and blue indicate northern edge sites.
